# An Exploration of Jamaican Mothers’ Perceptions of Closeness and Intimacy in the Mother–Child Relationship during Middle Childhood

**DOI:** 10.3389/fpsyg.2017.02148

**Published:** 2017-12-12

**Authors:** Taniesha Burke, Leon Kuczynski, Sonja Perren

**Affiliations:** ^1^Department of Psychology, University of Konstanz, Konstanz, Germany; ^2^Department of Family Relations and Applied Nutrition, University of Guelph, Guelph, ON, Canada; ^3^Department of Empirical Educational Research, University of Konstanz, Konstanz, Germany; ^4^Development and Education in Early Childhood, Thurgau University of Teacher Education, Kreuzlingen, Switzerland

**Keywords:** mother–child relationship, intimacy, Jamaica, closeness, middle childhood, parent–child relationships, parenting

## Abstract

Research on Jamaican mother–child relationships has had a limited focus on authoritarian parenting styles and selected discipline practices such as corporal punishment. This study examined Jamaican mothers’ experiences of closeness and connectedness with their children to provide a holistic perspective on Jamaican-parent–child relationships. Thirty mothers (17 middle class and 13 lower class) living in Kingston and St. Andrew, Jamaica, participated in a 1-h to 1.5-h semi-structured, open-ended interview regarding their 8- to 12-year-old children. Thematic analyses indicated that mothers experienced closeness through intimate interactions (e.g., shared projects, shared physical affection, mutuality, and child self-disclosure) and parent–child nurturance. Both mothers and children were active in creating contexts for closeness. Mothers also reported experiences that temporarily damaged their connection with their children. The findings suggest that the construct of parent–child intimacy may be useful in teasing out the psychological meanings and interpersonal processes of parent–child relatedness in cultural research.

## Introduction

Parents in all cultures love their children and have a strong psychological connection to them. However, the way this connection is expressed and communicated in the parent–child relationship depends on the relationship’s history and its cultural context (Trommsdorff and Kornadt, 2003). Interactions between parents and children occur in a distinctive long-term relationship context characterized by an immense history of interactions, a projected future, and close interdependence of their actions and emotions (Kuczynski and De Mol, 2015). These features of the relationship affect the dynamics of influence, connection, and vulnerability that occur between parents and children (Kuczynski, 2003). Moreover, the parent–child relationship is embedded in specific cultures that provide norms, values, and other meanings regarding power relations, and appropriate communication between parents and children (Trommsdorff and Kornadt, 2003; Trommsdorff, 2006). Cross-cultural studies suggest that although parent–child relationships have childrearing functions that are universal, the form that these functions take are adapted to specific needs, history, and settings of a given culture (Bornstein, 1995). In the Jamaican context, parent–child relationships have been shaped by a complex set of socioeconomic demographics, cultural, and religious influences that are a result of the island’s history of slavery, colonialism, and independence (Barrow, 2001).

Research on Jamaican families has explored unique family structures and childrearing practices that shape parent–child relationships. A predominant focus has been on parents in the role of disciplinarians and authority figures. Such studies suggest that during early childhood and adolescence, Jamaican parents use an authoritarian approach to childrearing that emphasizes hierarchical power in the family and obedience to authority (Crawford-Brown, 1999; Samms-Vaughan et al., 2005; Brown and Johnson, 2008). This authoritarian approach includes a pattern of frequent use of coercive discipline strategies including corporal punishment, shaming, and humiliation, which is present across the different socioeconomic classes (Smith and Mosby, 2003; UNICEF, 2010). This narrow focus on a single aspect of the parent–child relationships means that there is little understanding of how Jamaican parents engage positively with their children to foster close connectedness or create positive relational contexts for children’s development.

Contemporary perspectives on socialization (Maccoby, 2000; Grusec and Davidov, 2010; Kuczynski and De Mol, 2015) recognize that parent–child relationships are complex in their structure and in the nature of parent–child dynamics during social interactions. The parent–child relationship can be considered, holistically, as comprising three culturally embedded relational domains: the authority domain, the attachment domain, and the intimacy domain (Kuczynski and De Mol, 2015). These domains, which differ in their functions and power dynamics, emerge as parents engage in their various roles as socializing agents, as providers of protection and security, and as relationship partners striving to maintain a close personal connection with their children.

Parent–child intimacy and related concepts such as mutuality (Oliphant and Kuczynski, 2011), reciprocal interactions (Grusec and Davidov, 2010), warmth (Laible et al., 2014), and affiliation (Killen and Wairnryb, 2000; Rothbaum and Trommsdorff, 2007), have received increasing attention as a domain of the parent–child relationship, which is distinct from attachment (Grusec and Davidov, 2010; Kuczynski and De Mol, 2015). The intimacy domain concerns positive parent–child interactions that are experienced as mutually enjoyable for parents and children (Oliphant and Kuczynski, 2011). In contrast, the attachment domain is concerned with the parental provision of protection and security when children are distressed and in need of care (Grusec and Davidov, 2010).

Like attachment, mutually positive interactions are empirically associated with important socialization outcomes. Parental responsiveness to positive initiations by children has been found to promote children’s willingness to cooperate with parental requests and rules (Parpal and Maccoby, 1985; Kochanska, 1997). Moreover, frequent mutually positive interactions are associated with children’s development of conscience (Kochanska and Murray, 2000), internalization of values (Kochanska and Aksan, 1995), enhanced social skills (Lindsey et al., 1997), and children’s responsiveness to parents during social interactions (Kochanska and Murray, 2000).

Although positive experiences of parent–child relatedness are beneficial for children, the form that these experiences take is culturally specific (Kagitcibasi, 2005; Rothbaum and Trommsdorff, 2007). There is evidence that cultures differ in their norms for how intimacy is expressed between parents and children. Within individualistic societies, intimacy may be expressed in daily interactions through physical and emotional expressiveness, self-disclosure, and shared enjoyment (Derlega, 1984; Monsour, 1992). Research with middle class Canadian families (Harach and Kuczynski, 2005; Oliphant and Kuczynski, 2011) found that intimacy was the primary way that parents conceptualized strong parent–child relationships during middle childhood. Both mothers and fathers reported that they value intimate interactions, search them out, and strive to restore intimacy when tensions develop in the relationship after interactional missteps. In contrast, parents in collectivist societies may be more restrained in the communication of close relatedness, but demonstrate their love for children through self-sacrifice and meeting children’s needs (Lim and Lim, 2004; Rothbaum and Trommsdorff, 2007; Clayton, 2014).

The purpose of the current study is to explore the neglected domain of intimacy in Jamaican parent–child relationships. Research on parent–child relatedness is surprisingly sparse in the literature on Jamaican mother–child relationships. It is unclear how parent–child closeness is expressed and experienced in the Jamaican cultural context. Jamaica is classified as a collectivist culture (Hofstede, 2011) with evidence of hierarchical power relations, authoritarian parenting styles and harsh discipline (UNICEF, 2010). The strong emphasis on obedience, parental power, and a hierarchical form of social interactions may lead to the expectation that the culture is not conducive to parent–child closeness. Alternatively, it is possible that Jamaican parents may experience and express closeness in ways that differ from parents in Western individualistic societies (Trommsdorff and Kornadt, 2003). In an earlier study, Brown and Johnson () reported that Jamaican parents encourage hugs and praises during parent–child interactions. Furthermore, Ferguson and Iturbide (2015) suggested that traditional hierarchical patterns of interaction may be changing due to processes of acculturation because of contact with egalitarian American models of parent–child relationships. The authors found that American media influenced some mothers to encourage their children to self-disclose and respectfully voice their opinions.

It is also possible that social class may influence the form and frequency of intimate interactions. Social class is a crucial contextual factor for understanding parent–child relationships in Jamaican families. Lower class Jamaican parents are more likely to insist on compliance and obedience from their children (Anderson, 2007), and their verbal communications, tend to be confined to reprimands (Barrow, 1996; Brown and Johnson, 2008). Middle class parents encourage self-direction, assertiveness (Anderson, 2007), and open communication (Brown and Johnson, 2008). Therefore, the frequency and importance of intimate interactions are expected to be higher in middle-class families than in lower class families.

This study was guided by a specific theoretical conception of intimacy. Weingarten (1991, p. 294) defined intimacy as momentary interactions that occur “when people share or co-create meaning and are able to coordinate their actions to reflect their mutual meaning-making.” Weingarten also argued that the experience of intimacy may diminish when one person imposes meaning on the other or withdraws from meaning making. This is a cognitive conception of intimacy, that in the case of parent–child relationships, requires the perception by one or both partners in an interaction that they have participated in the co-construction of an event where there is the same understanding of the communication, emotion, or experience. Oliphant and Kuczynski (2011) found that this conception of intimate interactions was consistent with the mutually enjoyable interactions that Canadian parents reported as evoking the experience of closeness. For the present study, the construct of intimate interactions had the advantage of providing a specific conception of parent–child closeness which is comparable to the experiences of Jamaican parents. Additionally, the conceptualization of parent–child intimacy as co-constructed meaning provided a framework to ask theoretically guided questions concerning Jamaican mothers’ perceptions of their contributions to several linked processes including the construction of intimate experiences, the construction of non-intimate experiences, and relationship repair after interactional missteps.

As well, parents and children were conceptualized as being agentic actors who both contribute to the bidirectional dynamics of parent–child interactions (Kuczynski, 2003). Therefore, we were interested in mothers’ perceptions of their contributions as well as the contributions of their children to the formation of intimate interactions. The research strategy was to ask mothers to describe specific instances when they felt a sense of closeness with their children. The term “closeness” was used because parent–child intimacy is a label for a theoretical construct and may not correspond to the language used by mothers themselves for the same experiences. This approach allowed Jamaican mothers to define closeness in their own terms. Moreover, it afforded the possibility of understanding close connection in a culture-specific manner that may or may not correspond to the phenomenon of co-constructed intimate interactions found in Western families (e.g., Oliphant and Kuczynski, 2011).

## Materials and Methods

### Participants

Thirty mothers living in the urban areas of Kingston and St. Andrew, Jamaica, participated in the study. Their average age was 39.43 years (ranging from 29 to 50 years; *SD* = 5.57 years), and on average they had 2.5 children. **Table 1** contains the details of participants’ marital status, education level, employment status, and living arrangements. Participants were classified into middle class (17 participants, 56.67%) and lower class (13 participants, 43.33%). Social class was determined with the aid of the Statistical Institute of Jamaica 2011 census maps of enumerated districts, and participants’ demographic information. The combined information determined the communities that were known to have a history of violence and low income. The criteria for participation included Jamaican citizenship, residence in the Kingston and St. Andrew metropolitan area, and having at least one biological one child in the home who was between the ages of 8 and 12. There were no specific requirements for family structure. Thus, the final sample included mothers in married, common-law, and single parent family contexts. Each mother was asked to select one of their children who met the age criteria to be the focus of the interview. The final sample consisted of mothers’ interviews regarding 16 girls and 14 boys (*M* = 10.47 years, *SD* = 1.43). Eight girls and nine boys were from the middle class, and eight girls and five boys were from the lower class.

**Table 1:** Description of sample characteristics.

Variables	Middle class mothers (*n* = 17)	Lower class mothers (*n* = 13)
Marital status		
Married	14 (82%)	2 (15%)
Divorced	1 (6%)	0
Common-law	2 (12%)	5 (39%)
Single	0	6 (46%)
Education level		
University	16 (94%)	3 (23%)
Community college	1 (6%)	1 (8%)
HEART-NTA	0	2 (15%)
Other post-secondary	0	3 (23%)
Secondary	0	4 (31%)
Employment status		
Full-time	13 (77%)	6 (46%)
Part-time	3 (18%)	2 (15%)
Self-employed	1 (6%)	3 (23%)
Unemployed	0	2 (15%
Living arrangement		
Tenement yard	0	10 (77%)
Detached	17 (100%)	3 (23%)

*HEART-NTA means Human Employment and Resource Training – National Training Agency Certification. Other post-secondary includes accounting school, cosmetology school and university continuing studies. A tenement yard is a multi-family housing arrangement or compound consisting of several substandard dwellings on the same property called “the yard.” The yard usually consists of multiple families that are biologically related. There are occasions where non-biological families live on the same property. Tenement yards are situated in inner city communities, many of which are known as “garrisons.” Garrisons can be volatile with sporadic and unexpected moments of violence*.

### Procedure

University research ethics boards in Canada and Jamaica approved the study. A purposive sampling method was used to recruit participants. Local pastors, community leaders, human resource managers of companies located in Kingston and St. Andrew, and the authors’ contacts were solicited to help recruit participants. Local contacts received a flyer that contained details of the study’s purpose, requirements, and compensation. Participants granted informed consent before the commencement of the interviews. Each interview took place in mothers’ homes or a mutually agreed location and lasted between 1-h and 1.5-h. The interviews were recorded with a digital audio device and transcribed verbatim. Participants received a prepaid phone credit worth approximately US$2. The first author who is a native Jamaican and is fluent in the Jamaican dialect, Patois, conducted all the interviews. The first author built rapport with participants by disclosing her Jamaican upbringing and education, and her experience as a Jamaican parent. She conducted some of the interviews in the Patois dialect according to the mothers’ preference.

The Jamaican Family Relationship Interview Guide was used to conduct the interviews. The guide consisted of two parts. Mothers responded to open-ended questions regarding the parent–child relationship and the community context of childrearing. Mothers were then asked about specific domains of their relationship with the focal child using the critical incident technique (Butterfield et al., 2005). The critical incident technique is a procedure that asks informants to vividly recall recent specific incidents in their lives and to report the context, behaviors, emotions, and interpretations associated with the event. In this study, mothers were asked to recall in detail four recent incidents where they set and enforced rules for children (authority domain), four incidents where they responded to children’s distress (attachment domain), four incidents when they felt close to their children, and four incidents that adversely affected closeness (intimacy domain). This article only focuses on the data regarding the intimacy domain. For each incident that mothers described as involving an experience of close connection to their children, mothers were asked to describe who initiated the incident, what took place during in the interaction, and how they felt about interaction.

### Qualitative Thematic Analysis

Native Jamaican speakers transcribed the interviews to preserve participants’ original use of English or Patois. The first author conducted the analyses of transcripts using the interpretative phenomenology as the conceptual framework, and theoretical thematic analysis as the methodological framework for qualitative research. Interpretive phenomenology has the aim of describing and understanding the meanings of individual experiences within various social, cultural, psychological, and theoretical contexts (Larkin et al., 2006). Braun and Clarke’s (2006) theoretical thematic analysis guided the analysis procedurally. The transcripts were read multiple times, and memos were created to track significant events, ideas or concept and to ensure that the analysis did not prematurely constrain the interpretation of the data. Existing theory on parent–child relationships, and the first author’s knowledge of Jamaican culture provided sensitizing concepts that the researcher brought to the study (Kuczynski and Daly, 2003). Although these sensitizing concepts initially guided the analysis, they did not determine the final categories because the researchers were alert to novel concepts and themes in the participants’ narratives that were not present in the literature. Themes were refined, and interpretations were modified to ensure that they represented ideas communicated by the participants.

#### Trustworthiness

Several steps were taken to establish the trustworthiness of the analyses (Rolfe, 2006; Schwandt et al., 2007). Throughout the analysis, the researchers used MAXQDA (VERBI Software, 1989–2016), a software program that is designed to assist qualitative researchers with the systematic categorization of data and documenting the analytic process. This process provided an audit trail that included daily logs which recorded the process of theme development, analysis decisions, justification of final themes, and discussions related to theories and concepts that helped to explain the results (Lincoln and Guba, 1985). Additionally, an independent review of the analyses occurred during regular meetings with the second author who verified and challenged emerging interpretations of the data. These meetings ensured that the themes represented participants’ description of their experiences, increased the scope of the interpretations, and highlighted unique aspects of the culture that might have been missed by the first author. For example, the first author, who is Jamaican, took for granted the significance of the practice of situating multiple family homes on a single property known as a “yard.” However, the second author who was a cultural outsider considered these practices as examples of culture-specific communal living arrangements that have implications for socialization processes and the dynamics of family interactions.

## Results

Three major themes emerged from the analyses of mothers’ experiences of closeness in the mother–child relationship: (a) Jamaican mothers’ experience of closeness; (b) the context of mother–child intimate interactions; and (c) barriers to intimacy which emerged for mothers’ experiences of incidences that hindered mother–child closeness. The results include a description of each theme and their subthemes. Supporting quotes for each theme are presented in the original Jamaican dialect to maintain the authenticity of responses. The age and sex of the index child and the social class of the mother are used to identify each quote. Similarities and differences in the reported experiences between the social classes are highlighted qualitatively. A table is provided to indicate the percentage and frequency of themes expressed by middle class (MC) and lower class (LC) mothers. The small sample size did not permit quantitative analyses. Therefore, the frequencies across social classes do not represent statistical differences.

### Jamaican Mothers’ Experiences of Closeness

Jamaican mothers reported two qualitatively different experiences where they felt a close connection with their children. *Parent–child intimacy* captured mothers’ experiences in which they perceived that they engaged in interactions of mutuality with their children. *Parent–child nurturance* captured situations in which mothers felt closeness during interactions where they or their children provided care or comfort to each other.

#### Parent–Child Intimacy

Parent–child intimacy had four subthemes: (1) child self-disclosure; (2) shared positivity and enjoyment; (3) shared physical affection; and (4) shared projects. In each subtheme, mothers indicated that they perceived that both they and their children actively influenced the relational dynamics. Although mothers in both social classes expressed most subthemes, more middle class mothers reported intimate experiences than lower class mothers (**Table 2**).

**Table 2:** Themes in mothers’ experiences and contexts of closeness and barriers to non-closeness.

	Middle class (*n* = 17)	Lower class (*n* = 13)	Total (*N* = 30)
**Experiences of closeness**			
Parent–child intimacy			
Child’s self-disclosure	59% (10)	46% (6)	53% (16)
Shared positivity	100% (17)	85% (11)	93% (28)
Shared physical affection	65% (11)	39% (5)	53% (16)
Shared projects	35% (6)	15% (2)	27% (8)
Parent–child nurturance			
Child provides care	65% (11)	77% (10)	67% (20)
Mother provides care	51% (9)	62% (8)	57% (17)
**Contexts of closeness**			
Success and achievements	65% (11)	39% (5)	53% (16)
Leisure activities	53% (9)	46% (6)	50% (15)
Child-initiated intimacy	71% (12)	77% (10)	73% (23)
Family routines	94% (16)	31% (4)	67% (20)
**Barriers to closeness**			
Violations of expectations	77% (13)	46% (6)	63% (19)
Perception of rejection	29% (5)	31% (4)	30% (9)
Relational conflicts	35% (6)	15% (2)	27% (8)

*Table includes percentage of mothers. Absolute values are in parentheses*.

##### Child’s self-disclosure

Mothers reported feeling close to their children when their children disclosed their private thoughts, feelings, and personal information about mundane or significant aspects of their life during conversations. Child self-disclosure contributed positively to the dynamics of the relationship because it allowed mothers to gain insight into their children’s inner lives while providing the children with the opportunity to communicate what was important to them in an affectively positive social interaction. For example, “The moment of closeness is like when he is talking about something that might have happened at school or he is relaying some kind of experience” (MC: 12-year-old son). Or “He will share his thoughts about just any random thing.” “You know I saw this today?” “I don’t think that this makes sense.” Or “You know there is this joke I heard…” (MC: 8-year-old son).

##### Shared positivity and enjoyment

Most mothers from both social classes described interactions that included mutually shared positivity and enjoyment. Mothers’ narratives of mutual enjoyment often consisted of feelings of mutuality during conversations, mundane routines, and leisurely activities. For example, “We sit and watch TV and discuss the movie” (LC: 12-year-old daughter). Another mother talked about the personal significance she experienced as a mother during these conversations. “It’s just the joy, the joy, of having a child. When she is here, you know, you sit around the table and talk” (MC: 8-year-old daughter). Mothers also described experiencing emotions of pride, empathy, and shared joy when their children succeeded in the competitive context of the Jamaican education system. One mother recounted her experience when she received her child’s Grade Six Achievement Test (GSAT) results:

When she had passed GSAT, and I see how she put out so many Saturday classes, Sunday classes. Sleepless nights, sometime she go to her bed so late. She not even eat sometime because of the studying…When I heard the results I was at work…when the phone ring, and me see seh a the teacher a call, now you know me so scared to answer the phone so me give me co-worker the phone and said “Talk to him, talk to him.” Me hear she scream out and me just feel proud because even that day me cry, you know (LC: 12-year-old daughter).

##### Shared physical affection

Mothers reported that they felt a connection with their children when they shared physical affection. One mother reported: “He will come in the kitchen, and if I am cooking he come and hug me up and I will stop what am doing to hug him” (MC: 11-year-old son). Another mother described such contacts as an exclusive expression of affection that was particular to her relationship with one of her daughters, “Well most times she will just come and hug me up, you know. My other daughters don’t really do that, you know, but she do that” (LC: 12-year-old daughter).

##### Shared projects

Mothers described feeling closeness with their children when they engaged in shared projects together. These projects were activities in which children invited their mothers, or the mothers volunteered to join their children in a mutual task where the thoughts and actions of both partners were engaged in the co-construction of a common goal. Several mothers described that they experienced a special connection with their children when they assisted with their children’s academic studies. “I feel close to her when we are doing homework together in the evenings” (MC: 12-year-old daughter). Others spoke about the intimacy they experienced when they worked together on household chores. “We would be in the kitchen together, she would assist me, she is very helpful…We would do the laundry together, and she helps me to pin clothes on the line and stuff like that” (LC: 11-year-old daughter). Many mothers also described feeling close to their children when they engaged together in spiritual activities. One mother said, “We always read together. We read devotion, we always read the bible and explain it to them” (LC: 10-year-old son). Shared religious activity may be an example of a cultural context of parent–child intimacy because many Jamaican parents in our sample proudly proclaimed their religious observance.

#### Parent–Child Nurturance

Unlike the theme of intimacy which involved a sense of close connection during mutually enjoyable interactions, the theme of nurturance captured mothers’ experiences of closeness during complementary interactions when one person in the relationship was vulnerable or distressed and the other provided care or comfort. Most Jamaican mothers from both social classes reported experiencing closeness with their children during situations where they were either the recipient or initiator of caregiving.

##### Child providing care

Most mothers reported that they felt a sense of closeness when their children came to their aid and attempted to alleviate their distress, hurt, or frustration. One mother described the poignant connection she felt when she realized that she mattered to her child during a time that the mother was emotionally overwhelmed. She said,

There was one particular incident…where I was actually in for a job and because of some paper works…the offer was rescinded, and I felt really crushed because…I had just resigned from the other job…So in that period…I had no income and like I was depressed…and she just came to me and she said “mom…I hear you crying…” she hugged me and say it’s going to be okay, and she pat my head like it’s going to be fine. You’re my mommy. You always take care of me, so it’s okay (MC: 9-year-old daughter).

Mothers also reported that they felt connected to their children when their children commiserated with them and provided sensitive care when mothers were ill or in distress. These interactions communicated to mothers that they mattered to their children and promoted an experience of emotional connection.

When I said I was sick…I had to do surgery, and I took out all four of my wisdom tooth at one time…there was a time when I was just in the room and lying down and the pain was so bad… he just came up he saw and he was like “Oh my goodness it is really bad” and tried to make me comfortable (MC, 9-year-old son).

Unique to our lower class sample were several reports of mothers who said they felt cared for when their children comforted them when their partners/spouses behaved coercively. “On a regular basis when his father disappoint mi an him seh mommy a soon leave school enuh and going to work and you’re going to be fine” (LC: 10-year-old son). Another mother described how her child consoled her and offered assurances of security when she lost custody of her other child, “I guess when my 4-year-old daughter left and I was crying for weeks and days and weeks and days. She was always there saying, “Mommy I love you and you know I am not going to leave you. And always a kiss-kiss me up” (LC: 10-year-old daughter).

##### Mother providing care

Most mothers also reported that they felt close to their children when they provided comfort to their children during moments of vulnerability resulting from illness or distress. Mothers who reported these experiences discussed the importance of being there when their children needed comforting.

When he had his surgery…he had…his tonsils removed and he didn’t want, like everybody else was in the hospital. But like I had to stay, and I had to be on the bed with him. It was fine for everybody else to be in the room, but I had to be right beside him (MC: 8-year-old son).

Mothers reported feeling close to their children when they had to provide emotional support during moments when their children failed at achieving academic expectations. “When she do her GSAT. She didn’t get the school of her choice and she was very sad, so I didn’t want it to mess up her brain or anything so I got really emotionally close to her that time” (LC: 12-year-old daughter).

### Contexts of Mothers’ Intimate Experiences

Mothers reports also provided insights into the specific situations that provided opportunities for co-constructing moments of mutuality. Mothers reported four contexts of intimate interactions: *success and achievements, leisure activities, child-initiated intimacy, and family routines.*

#### Success and Achievements

Because educational achievement is one of the leading avenues for social mobility in Jamaica, the Jamaican school system is very competitive. Many parents reported that their children’s academic successes served as a context for shared enjoyable moments in the relationship. One mother described how her child’s achievement was the context of intense pride and mutual joy. “At school at the end of the school year when we had prize giving and just to hear his name being announced for the awards that he is getting. It, you know, I really feel that sense of like wow! Umm, my heart!” (MC, 10-year-old son). A lower class mother also reported a close connection to her son when he joyfully announced his good grades, “If he did something good at school, like he achieves his goal in the maths or anything with a pass mark really good he would come home and be so excited (LC: 11-year-old son).

#### Leisure Activities

Leisure was reported to be a context for shared enjoyable interactions by half the sample. Middle class mothers described leisure activities as traveling and shopping, regular dates, and mundane events outside the home. One mother described an example of intimate interactions while traveling with her child, “My daughter and I took a trip to Florida…So, we were away from Daddy for a few weeks. So, it was just my daughter and I everywhere, so you know, so just with the time alone with her” (MC: 8-year-old daughter). Leisure activities for lower class mothers were almost exclusively activities within the home: “We watch TV together” (LC: 12-year-old son).

#### Child-Initiated Intimacy

Interactions, where children were the agents and initiators of intimate interactions, were particularly meaningful for mothers. Mothers not only felt close to their children when their children drew them into an intimate interaction. They also experienced their children’s desire to engage with them as a tangible sign that they mattered to their children in the relationship. Children’s voluntary self-disclosures became the context of mutually enjoyable interactions. “She just crawled in bed, and I was lying in bed.” I remember distinctly, and she said, “Mummy you know what? My period is here” (MC, 12-year-old daughter). Another mother reported her daughter offered her a meal. The theme was also related to the context of mutually shared positivity. “She also prepares her meal herself, so then sometime she will come and say mommy you want me to make pancake? And she will ask and say, mommy can you make French toast?” (MC, 11-year-old daughter). Mothers also described feeling deep a connection when their children spontaneously expressed physical affection to them, “I will be sitting there and him come…mommy a wah talk to yuh an him put him head inna yuh lap” (LC: 10-year-old son).

#### Family Routines

Middle class mothers frequently reported moments of intimacy that happened in the contexts of everyday family routines. Family routines included bedtime routines, morning routines, daily tasks, and transportation. Examples of mutuality in shared positivity include bedtime routines, “Well, I guess number one is bedtime routine when I do have the energy, but it happens more often than not, the reading and putting them to bed” (MC, 9-year-old daughter). Morning routines also involved mutually shared positivity and enjoyable experiences. “In the morning, depends on who sends him off, we pray together” (MC, 10-year-old son). Daily tasks such as chores or homework served as a context of shared pleasurable moments, “It’s like when he carries out his responsible chores. When he gets involved and he takes up initiative to do something in the house” (MC, 10-year-old son). Another mother said, “Doing homework. Most times homework is a family thing” (MC, 8-year-old daughter). Transportation routines during which mothers and children were confined in close quarters of a car provided an opportunity for them to connect with their children. “When I pick her up from school, we will have our chit-chat about how your day was, and she tell you who trouble her from who don’t trouble her” (MC, 12-year-old daughter). Departures also provided occasions for positive interactions that elicited a moment of closeness. “When he leaves out the car and a say be good, and he say ‘bye’ mommy a feel close to him” (LC: 11-year-old son).

### Non-intimate Interactions: Barriers to Intimacy

Jamaican mothers’ descriptions of occasions when they experienced diminished feelings of close connection with their children provided insights into the barriers to parent–child intimacy. The three themes that emerged: *violation of parental expectations, conflict, and perception of rejection* (see **Table 2**) can be interpreted as occasions when the perception of mutuality is contradicted by the behavior of one or both partners in an interaction.

#### Violations of Parental Expectations

Most mothers in our sample perceived that their feelings of closeness were diminished when their children’s behavior was not consistent with mothers’ character goals for their children or when their children did not comply with specific demands. One mother described her disappointment when her child was not pushing himself to be better academically. She said, “I don’t feel really close to him is that when I know he has the potential to do better and he doesn’t do it” (MC: 10-year-old son). Other mothers described feeling distance in the relationship when their children transgressed against mothers’ expectations for prosocial behavior such as kindness to others. “When he is being selfish, hard for me to tolerate” (MC: 9-year-old son). One mother interpreted her child’s failure to disclose a personal secret as a contradiction to her belief that their relationship allowed for open communication. “When I don’t feel close was like the other day I was feeling a little down because her period started, and she didn’t say anything” (LC: 12-year-old daughter). Mothers also reported that non-compliance with parental requests caused temporary tensions in the parent–child relationship. One mother said, “Of course, when he is being disobedient and wants to have his own way” (MC: 10-year-old son).

#### Conflict

Mothers perceived the occurrences of conflict, differences in opinion, or correcting children for misbehaviors as detrimental to intimacy. Mothers regarded such conflicts as non-mutual. One mother described disagreements as diminishing closeness, “I guess the natural thing to say is when we disagree we are not close” (MC: 11-year-old son). Interactions, where mothers punished or corrected their children, were also perceived as disrupting a sense of mutuality and closeness. “When we don’t seem close is usually when she doesn’t get her way and I have to discipline her. And she just thinks I’m being too strict or I’m being over bearing” (MC: 9-year-old daughter). Another mother reported a similar experience: “When I have change out the gear and get really stern at him and punish him” (LC: 12-year-old son).

#### Perception of Rejection

A third of our participants reported that they did not feel close to their children when they interpreted their behavior as relational rejection. One mother felt rejected when she perceived that her child turned to another relative for care and security. “When she is ill she trusts my brother” (MC: 9-year-old daughter). Another mother felt rejected when her child withdrew from communicating with her after a reprimand. “Well if she wants to go somewhere and I tell her no she vexed with me for the whole time, probably won’t talk to me” (LC: 12-year-old daughter).

## Discussion

This study provided insight into a neglected aspect of Jamaican parent–child relationships by adding to an abundant literature on parental discipline and control, a balancing exploration of parent–child connection, and intimacy. Empirically, the findings suggest that Jamaican mothers engaged in and valued intimate social interactions with their children, which was characterized by positive mutuality, openness, emotionally significant feelings of relatedness, and mattering. Jamaican mothers implicitly conceptualized closeness with their children in two qualitatively different ways – intimacy and nurturance.

Mothers’ reports of mother–child connection in the context of interactions where they perceived mutuality with their children were consistent with Weingarten’s (1991) conception of intimacy as transient interactions, characterized by co-constructed moments of shared meaning. For mothers, intimacy occurred when they perceived that they and their children engaged in coordinated mutually enjoyable moments of connection. These interactions included shared positive emotions, engaging in shared projects, conversations in which children disclosed their inner life, and shared physical affection. Mothers’ perceptions of non-intimate interactions such as expectancy violations, conflict, and rejection are also consistent with Weingarten’s argument that withdrawal from mutual meaning-making or imposition of meaning by one or both partners is a barrier to intimacy.

The fact that Jamaican mothers experienced close connection in the context of intimate mutual interactions in a manner similar to findings for Canadian families (Harach and Kuczynski, 2005; Oliphant and Kuczynski, 2011) was unexpected. A background assumption of this study was that the collectivistic values within Jamaican culture and evidence of harsh authoritarian parenting practices found in previous research would mitigate against intimacy as a way of expressing or experiencing close connection in the parent–child relationship. Given that the expression and communication of relatedness have been understudied in cross-cultural research, many questions remain for the future. Perhaps the construct of parent–child intimacy, which depends more on the perception of mutuality than on the open expression of affection, is a general process of connection in parent–child relationships across cultures.

Alternatively, the finding that mothers perceived that both they and their children initiate and maintain shared positive interactions can be interpreted as resulting from norms of reciprocity prevalent in collectivistic cultures. In collectivistic cultures, children are socialized to understand that the support that they receive is a payoff that must be reciprocated (Twum-Danso, 2009). In the case of Jamaican parent–child relationships, the obligation to reciprocate may contribute to positive moments that may offset the adverse effects of harsh punishment that occur in other domains of the relationship.

Although the qualitative nature of the data precludes inferences of differences between the classes, indications that middle class mother reported intimate interactions more frequently, and a wider array of intimate experiences than lower class mothers is suggestive and needs further research. A potential reason for a difference between social classes is that middle class mothers may have more time and resources to dedicate to one-on-one interactions with their children than their lower class counterparts. Alternatively, children’s willingness to self-disclose to mothers may be related to findings that middle class parents engage in more open communication and encourage children to be assertive and outspoken (Anderson, 2007; Brown and Johnson, 2008). Another explanation for the findings could be the living arrangement of the families in our sample. Most of the families in the lower class lived in tenement yards with multiple extended family dwellings rather than the single-family houses of the middle class participants. In the tenement yard context, children readily have access to multiple adults with whom they can experience intimate interactions. The availability of these alternative close relationships may reduce the frequency of intimate interactions with mothers. More research is needed to determine the effects of communal living on the type, quality, and frequency of parent–child intimacy.

The finding that Jamaican mothers also felt close to children in the act of giving and receiving nurturance during times of stress and vulnerability is also important to highlight as a new contribution to the literature on parent–child relationships. Theoretically, the processes involved in nurturing interactions are distinct from the processes underlying the perception of enjoyable mutuality that underlies the theory of parent–child intimacy. Mothers reported that they felt close to their children and experienced as a sense of mattering to their children when they either provided care to their children or received care from them. Mattering is “the psychological tendency to evaluate the self as significant to special other people” (Marshall, 2001, p. 474). Mothers’ description of nurturance as a form of closeness is interesting and novel because it has a different power dynamic than the construct of parent–child intimacy. Conceptually, the bidirectional dynamics resemble the complementary power relations of the attachment domain. Kuczynski and De Mol (2015) argued that in the attachment domain, the goal is to provide security through responsive care when the recipient is vulnerable and in need of protection. In nurturing interactions, the dynamics consist of complementary power, such that one person displays a need for care and the other provides care.

The prevalence of reports that children provided emotional support and comfort to their mothers in our Jamaican sample needs further research. In particular, the narratives of lower class mothers are consistent with studies that have found that children’s caregiving was more prevalent in single-parent families, environmentally stressful conditions, and in contexts where parents were overtaxed (Barnett and Parker, 1998; Chase, 1999; Jurkovic et al., 2001; Mayseless et al., 2004). The meaning of this phenomenon is under debate. In the clinical literature, children’s caregiving has been conceptualized in a problem-focused way as “role reversal” or “parentification” (Boszormenyi-Nagy and Spark, 1981) because it implies that children are assuming caregiving roles that are usually ascribed to adults. The argument has been that children’s well-being may be adversely affected because they prematurely take on parental roles and responsibilities before they are developmentally equipped to do so (Boszormenyi-Nagy and Spark, 1981). However, some researchers have argued that the phenomenon of young children intervening to provide nurturance to their distressed parent is a constructive act of children’s agency (Burnett et al., 2006; Chee et al., 2014; Katz, 2015). In this interpretation, Jamaican mothers’ feelings of close connection may be derived from their perceptions that their children are voluntarily taking responsibility and acting to the best of their ability on behalf of a relationship in which they have a mutual stake.

Mothers attributed the temporary lack of closeness with their children to their perceptions of child rejection, mother–child conflict, and children’s violation of parental expectations. Middle class mothers reported a loss of closeness more than lower class mothers when their children violated expectations. It is possible that lower class mothers may not have experienced the same frequency and intensity of loss of closeness because of their communal environment in which all adults share in creating close interactions with children. It is important to note that the findings highlight the emotionally adverse effects of temporary loss of closeness among mothers of both social classes, which illustrates the value and importance they placed on these interactions.

The findings make significant contributions to the Jamaican literature and parent–child relationships in general. For instance, the evidence of closeness and positivity despite the presence of authoritarian parenting practices suggests a culturally constructive balance of authority, reciprocity, and relatedness. This observation indicates that the traditional western concept of authoritarian parenting may be inadequate in describing important nuances of parent–child relationships in collectivistic cultures such as Jamaica. As a direction for future research, there need to be more studies on the interplay between closeness and cultural expectations of interactions.

The emphasis on middle childhood period of development in this study is of benefit to the literature because middle childhood is an understudied developmental period in Jamaican research. Moreover, the findings may contribute to a better understanding of how changes in children’s cognitive abilities and social skills during this developmental stage influence parents’ perceptions of their children’s contributions as agents in family dynamics. Also, in examining middle childhood researchers may learn how closeness is maintained, evolved or transitioned between parents and children during adolescence and beyond.

These initial findings on the nature of close connections between Jamaican mothers and their children at middle childhood have implications for research and practice. It provides an entry point toward a more comprehensive understanding of Jamaican children’s socialization experiences. Much of the research in the Jamaican context has focused on parental discipline and control strategies as the principal vehicle for children’s socialization. However, there is growing evidence that socialization is also accomplished as parents engage in, and maintain mutually responsive parent–child relationships. In this relational perspective, experiences that build close connection, mutual positivity, and responsiveness are not a frill but are critical to understanding the foundations of children’s cooperation and positive social development (Kochanska et al., 2005; Aksan et al., 2006; Kuczynski and De Mol, 2015).

The findings add new questions and directions for research on relational dynamics of parent–child interactions in the Jamaican context. Many of the incidents reported by mothers involved a process of bidirectional influence between parents and children on a behavioral and cognitive level. Mothers perceived children as drawing them into interactions that mothers experienced as enjoyable and meaningful for their relationships, and mothers reciprocated in a positive way that was enjoyable for children. The findings support previous research on parent–child intimacy (Oliphant and Kuczynski, 2011), which argued that both parents and children are agents who contribute to the construction and maintenance of intimate interactions. More generally, such findings are consistent with contemporary models of socialization processes that emphasize bidirectional influence and interdependence in the parent–child relationships (Kuczynski, 2003).

Furthermore, by viewing parent–child intimacy as part of a more complex domain view of parent–child relationships raises questions about how the dynamics in the intimacy domain influence the behaviors and expectations of parents and children in the authority and attachment domains. For example, it is possible that enhancing positive mutuality in the intimacy domain may promote children’s cooperation with parental socialization efforts. As well, parental goals that focus on mutually enjoyable interactions may constrain parents’ use of coercive power in the authority domain or offset damages to the relationship when parents do overpower children with coercive control (Harach and Kuczynski, 2005).

Clinicians who work with families that may be experiencing challenges in parent–child relationships may find in the present research a way of communicating alternatives to coercive discipline. Moreover, intervention and preventative programs may demonstrate the positive role of intimate mutually enjoyable interactions, and promote awareness of various ways that parent–child mutuality can be enhanced to promote enjoyable and meaningful personal relationships, which benefit both parents and children.

### Limitations

This study had several limitations. The study used a single informant method, including only Jamaican mothers, which results in an incomplete picture of mother–child intimacy. Children may have different perspectives of interactions in the intimacy domain. Also, the qualitative nature of the study prevented comparisons between mothers’ experiences with sons and daughters. Future research may need to consider children’s gender when investigating mother–child intimate interactions. Mothers’ selection of one child may not have been random, which it may indicate that mothers selected the child they felt closest. Last, the study did not investigate father–child relationship. Previous studies have shown that the relationship that a child has with his/her mother and father is different (Bradley et al., 2015), which means that the examination of father–child relationships can provide a holistic picture of the Jamaican parent–child relationship.

## Author Contributions

TB conceptualized, planned, and conducted the research project. TB also analyzed the data and wrote the paper. LK supervised the execution of the project, independently reviewed the results, and revised the draft versions of the paper. SP provided feedback on the definition of the final results and revised draft versions of the paper. All authors read and approved the final manuscript.

## Conflict of Interest Statement

The authors declare that the research was conducted in the absence of any commercial or financial relationships that could be construed as a potential conflict of interest.
